# Taking the STING out of acute myeloid leukemia through macrophage-mediated phagocytosis

**DOI:** 10.1172/JCI157434

**Published:** 2022-03-01

**Authors:** William Brian Dalton, Gabriel Ghiaur, Linda M.S. Resar

**Affiliations:** 1 Department of Oncology, Johns Hopkins University School of Medicine, Baltimore, Maryland, USA.; 2 The Sidney Kimmel Comprehensive Cancer Center at Johns Hopkins, Baltimore, Maryland, USA.; 3 Department of Medicine, Division of Hematology,; 4 Department of Pathology, and; 5 Institute for Cell Engineering, Johns Hopkins University School of Medicine, Baltimore, Maryland, USA.

## Abstract

Macrophages within the bone marrow (BM) microenvironment take on unexpected roles in acute myeloid leukemia (AML) as reported by Moore and colleagues in this issue of the *JCI*. In contrast to solid tumors, where tumor-associated macrophages frequently assume an immunosuppressive phenotype that promotes tumor progression, this study revealed that BM macrophages repressed leukemia expansion in AML through a pathway called LC3-associated phagocytosis (LAP). After phagocytosis of dead and dying leukemic cells, including the mitochondria within the leukemic blasts, mitochondrial DNA activated stimulator of IFN genes (STING), leading to inflammatory signals that enhanced phagocytosis and restrained leukemic cell expansion. These findings unveil the modulation of macrophage-mediated phagocytosis via LAP as a potential therapeutic strategy directed at the BM microenvironment in AML.

## The microenvironment in regulating tumor progression

While increasing evidence underscores the key role for the microenvironment in tumor initiation, maintenance, and response to therapy in hematologic malignancies, studies elucidating actionable mechanisms within the tumor microenvironment (TME) in leukemia are only beginning to emerge (1–5). Earlier research on immune cells and adaptive immunity led to the development of chimeric antigen receptor (CAR) T cells, which revolutionized therapy for a subset of patients with acute lymphoblastic leukemia (6). However, a major hurdle to developing effective CAR T cells for acute myeloid leukemia (AML) is the paucity of leukemia-specific antigens (7). Recent studies focused on innate immunity revealed that delayed reconstitution of NK cells during allogeneic bone marrow (BM) transplantation correlates with relapse in patients with AML (8). Further, infusion of ex vivo–expanded NK cells together with the allograft improves outcomes (9). As demonstrated by Moore et al. (10), another exciting therapeutic strategy centers on modulating macrophage activity within the leukemia TME. In solid tumors, tumor-associated macrophages (TAMs) are composed of different cell populations that can either repress or foster tumor growth (11, 12). Consisting of at least two distinct subtypes, M1 TAMs exert antitumor effects through antibody-dependent phagocytosis of tumor cells, whereas M2 TAMs promote tumor progression by inhibiting T cell–mediated antitumor activity (11, 12). Leukemic cells evade macrophage-mediated phagocytosis by upregulating CD47, a cell surface receptor that signals “don’t eat me” to phagocytic macrophages (13). This discovery led to anti-CD47 antibody–mediated therapy, which is currently in late-stage clinical trials with promising results. The discovery of CD47 and subsequent clinical trials established the importance of macrophages, not only in tumor progression, but also in responses to antibody-based therapies (13). However, substantial gaps in our understanding of mechanisms controlling macrophage activity in AML remain, and the work by Moore et al. illuminates a surprising mechanism with potential for therapeutic opportunities (10).

## Mononuclear cell phagocytosis by LC3-associated phagocytosis

Over a decade ago, researchers discovered a form of phagocytosis that promotes optimal phagocyte maturation and degradation of ingested cargo using components of autophagy machinery (14). Because this process recruits the microtubule-associated protein light chain 3 (LC3) to the phagosome, it was denoted LC3-associated phagocytosis, or LAP (15). LAP contributes to the clearance of foreign pathogens or dying host cells, both of which trigger the process by engaging the T​ cell membrane protein 4 (TIM-4) receptor on the surface of macrophages (15). Accordingly, defects in LAP cause incomplete degradation of phagocytosed cargo, elevation of proinflammatory cytokines, and promotion of a systemic lupus erythematosus–like autoimmunity in mouse models (16). In murine models of solid tumors, such as melanoma and lung carcinoma, while defective LAP impairs the degradation of engulfed tumor cells, it stimulates antitumor immunity through the activation of tumor-infiltrating lymphocytes and the secretion of inflammatory mediators downstream of the stimulator of IFN genes (STING) pathway (11). Together, these findings suggested that LAP mediates immune suppression and tolerance, while defective LAP function could lead to autoimmune disease or defective tumor clearance (15, 16). In this issue of the *JCI*, Moore and authors investigated the role of LAP within the BM microenvironment in AML and uncovered another LAP function (10).

## LAP restricts AML expansion

Prior studies showed that depleting macrophages in vivo via the bisphosphonate clodronate expands the leukemic burden in both immunocompetent (*MLL-AF9*; ref. 17) and immunocompromised (18) models of AML, although the underlying mechanisms remain incompletely understood. Moore et al. confirmed that clodronate depleted macrophages within the BM microenvironment and increased the leukemia burden in two additional models of murine AML (MEIS1/HOXA9 and MN1 AML; ref. 10). The authors went on to show that BM macrophages from mice with AML had increased phagocytic activity compared with macrophages derived from BM of healthy mice. They also found that LC3 was recruited to the phagosome in AML, implicating the noncanonical phagocytosis pathway, LAP, in modulating the AML microenvironment. Using an elegant genetic approach, the authors demonstrated that LAP was the primary mechanism utilized by AML-associated macrophages for phagocytosis. They studied *Atg16L1^E230–^* mice rendered deficient in LAP by Cre-mediated excision of the linker and WD domains of Atg16L1 in macrophages. Importantly, both the linker and WD domains were required for LAP, but not for canonical pathway phagocytosis. Mice deficient in LAP had an increased tumor burden and shortened survival compared with LAP-proficient mice, in which macrophages were able to clear apoptotic bodies and apoptotic cells from the BM of mice with AML. Thus, in a microenvironment in which LAP was disrupted, the subsequent accumulation of AML apoptotic bodies promoted tumor growth. The immunologic mechanisms by which apoptotic debris promotes the expansion of leukemic cells, in contrast to its antitumor activity in models of solid tumors, remain incompletely understood (11). One potential clue was the observation that LAP deficiency resulted in the recruitment and activation of cytotoxic T cells in solid tumor models, which could restrain tumor expansion (11). In the study by Moore and colleagues, LAP deficiency increased CD4^+^ Th cells in one AML model (MN1), although, unlike prior solid tumor studies, there was no recruitment of cytotoxic CD8^+^ cells (10). The authors also found that AML cells promoted the expansion of both tumor-associated macrophages and resident BM macrophages, and neither of these processes was altered when LAP was impaired (10).

## LAP stimulates STING in BM macrophages

**Figure 1:**
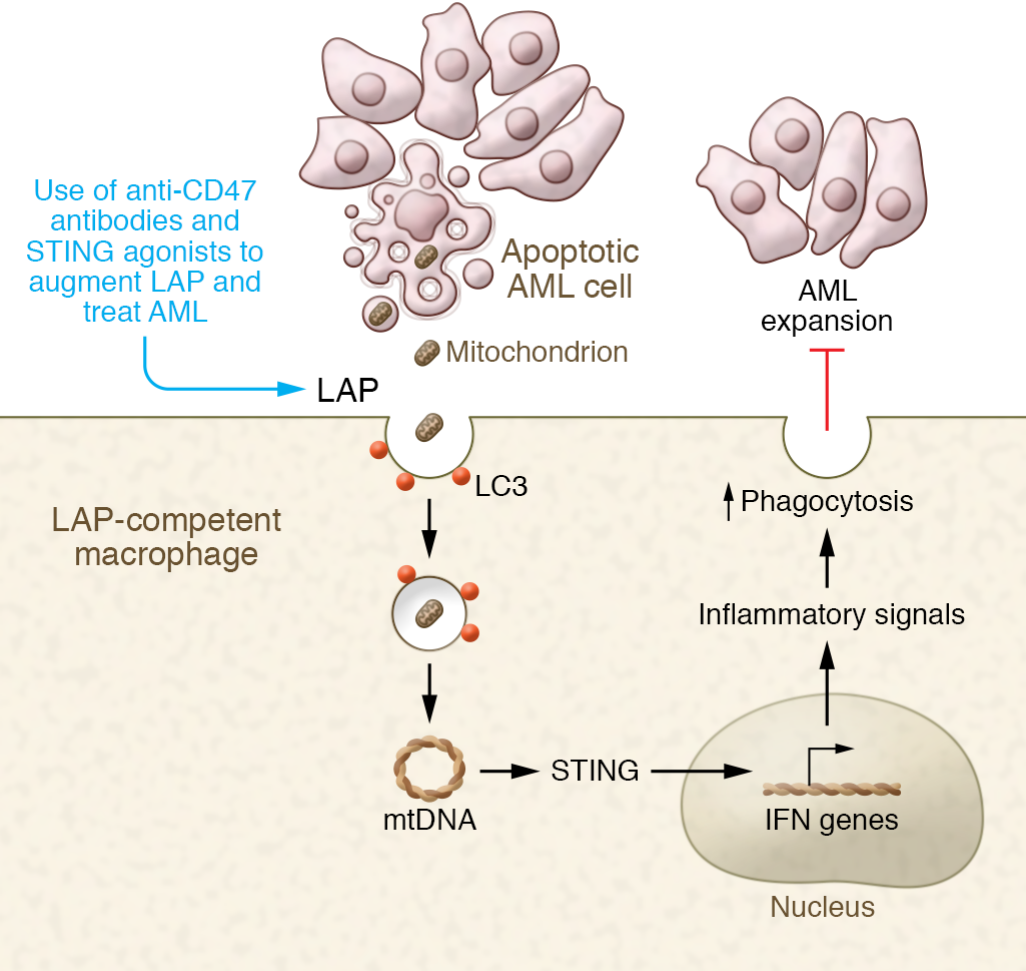
LAP-mediated phagocytosis by BM macrophages represses leukemic cell expansion in AML. Apoptotic bodies from AML cells with abundant mitochondria are ingested by BM macrophages via LAP, after which mtDNA stimulates STING to enhance phagocytosis and restrain AML cell growth. Future therapies to enhance LAP-mediated phagocytosis could have therapeutic benefit.

Turning to the molecular mechanisms by which LAP restricted AML growth, Moore and authors discovered that macrophages from LAP-competent *Atg16L1^E230+^* mice activated STING, as evidenced by increased expression of the IFN genes *Gbp2*, *Irf7*, and *Ifit3* (10). In contrast, LAP-incompetent *Atg16L1^E230–^* macrophages failed to activate STING. Furthermore, pharmacologic antagonism of STING with H-151 decreased IFN gene expression and directly suppressed the phagocytic activity of LAP-competent macrophages. Inhibition of both LAP and subsequent STING-mediated phagocytosis increased the AML tumor burden and shortened survival in the AML models. Building on the previous observations that AML cells have increased mitochondrial mass compared with that of nonmalignant cells (19), the authors directly tested whether mitochondria are involved in stimulating STING activation in LAP-competent macrophages. Notably, apoptotic bodies derived from AML cells contained increased mitochondrial content. Further, LAP-incompetent macrophages could not deliver apoptotic bodies to lysosomes for degradation. In an elegant twist, the authors then used the same AML cells, but devoid of mitochondria (generated by ethidium bromide treatment and designated as ρ0), to demonstrate that STING could not be activated when LAP-competent macrophages phagocytized the ρ0 derivatives (lacking mitochondria). From these findings, the authors concluded that, in contrast to LAP’s immunosuppressive role in murine models of solid tumors (11), LAP harnessed leukemic mitochondria to activate STING, stimulate further phagocytic potential, and inhibit the growth of leukemic blasts within the BM (Figure 1).

## Remaining questions and clinical translation in AML

While immune checkpoint inhibition revolutionized therapy for a subset of tumor types, immune-based therapy for AML has been limited. Recent strategies include manipulating immune cell populations in transplantation settings (8), targeting immune evasion signals (13), or, more recently, modulating immune signals within leukemic blasts (20). Importantly, outcomes for AML remain poor, particularly for older patients who frequently cannot tolerate intensive cytotoxic therapy or the preparative regimens required for BM transplantation (13, 20, 21). Further, the incidence of AML is expected to rise as our global populations age. Moore et al. revealed an interesting immune cell phagocytic pathway, LAP, active in AML-associated macrophages, that serves to restrain tumor growth (10). This AML-associated macrophage function contrasts with the function of M2 macrophages in solid tumors, which suppress antitumor immune responses. The researchers also uncovered another distinction between immune signaling in AML and solid tumor microenvironments. In AML-associated macrophages, STING was activated by phagocytosed mitochondrial DNA (mtDNA), which led to inflammatory signals that repressed leukemic cell expansion in AML without recruiting or activating cytotoxic T cells. By contrast, STING activation in solid tumor models recruits cytotoxic T cells that also restrain tumor growth (11). Further studies are needed to ascertain why these differences occur in the TME of AML compared with that of solid tumors. On the basis of their unexpected findings, however, the authors proposed that therapeutic interventions to enhance phagocytic macrophages within the AML microenvironment could be deployed to repress leukemic cell growth. Further studies are also needed to ascertain whether BM macrophages can adopt an M2 immunosuppressive phenotype and why macrophage expansion occurs without recruitment of T cells following STING activation in these AML models. Despite these unanswered questions, this work brings us closer to identifying immune mechanisms that could be modulated in therapy to improve outcomes for patients with AML.

## Version 1 03/01/2022

electronic publication
